# Coronary Artery Remodeling in a Model of Left Ventricular Pressure Overload Is Influenced by Platelets and Inflammatory Cells

**DOI:** 10.1371/journal.pone.0040196

**Published:** 2012-08-20

**Authors:** Fanmuyi Yang, Anping Dong, Paul Mueller, Jessica Caicedo, Alyssa Moore Sutton, Juliana Odetunde, Cordelia J. Barrick, Yuri M. Klyachkin, Ahmed Abdel-Latif, Susan S. Smyth

**Affiliations:** 1 Division of Cardiovascular Medicine, Gill Heart Institute, University of Kentucky, Lexington, Kentucky, United States of America; 2 Department of Genetics, The University of North Carolina at Chapel Hill, Chapel Hill, North Carolina, United States of America; 3 Lexington VA Medical Center, Lexington, Kentucky, United States of America; University of Padova, Italy

## Abstract

Left ventricular hypertrophy (LVH) is usually accompanied by intensive interstitial and perivascular fibrosis, which may contribute to arrhythmogenic sudden cardiac death. The mechanisms underlying the development of cardiac fibrosis are incompletely understood. To investigate the role of perivascular inflammation in coronary artery remodeling and cardiac fibrosis during hypertrophic ventricular remodeling, we used a well-established mouse model of LVH (transverse aortic constriction [TAC]). Three days after pressure overload, macrophages and T lymphocytes accumulated around and along left coronary arteries in association with luminal platelet deposition. Consistent with these histological findings, cardiac expression of IL-10 was upregulated and in the systemic circulation, platelet white blood cell aggregates tended to be higher in TAC animals compared to sham controls. Since platelets can dynamically modulate perivascular inflammation, we investigated the impact of thrombocytopenia on the response to TAC. Immunodepletion of platelets decreased early perivascular T lymphocytes' accumulation and altered subsequent coronary artery remodeling. The contribution of lymphocytes were examined in Rag1^−/−^ mice, which displayed significantly more intimal hyperplasia and perivascular fibrosis compared to wild-type mice following TAC. Collectively, our studies support a role of early perivascular accumulation of platelets and T lymphocytes in pressure overload-induced inflammation.

## Introduction

Left ventricular hypertrophy (LVH) is a common risk factor for the development of heart failure and an independent predictor of cardiovascular death. LVH usually develops in the setting of mechanical stress, overactive sympathetic drive, or as a consequence of genetic abnormalities. One important pathophysiologic characteristic of hypertensive LV remodeling is the production of excessive interstitial fibrillar collagen by fibroblasts [1]. Extensive fibrosis is thought to impair normal LV diastolic function and oxygen diffusion, leading to LV dysfunction [2]. Recent clinical studies have found a strong correlation between potentially fatal ventricular arrhythmias and myocardial fibrosis, as detected by delayed hyper-enhancement magnetic resonance imaging in patients with hypertrophic cardiomyopathy [3]. In addition to interstitial fibrosis, perivascular fibrosis occurs in the setting of LVH and congestive heart failure (CHF). LVH also results in susceptibility to myocardial hypoperfusion and ischemia as a result of disturbances in coronary flow physiology, that likely reflects remodeling of coronary arteries [4]–[9].

Perivascular inflammation has been observed in hypertensive disease and CHF and may initiate fibrosis and coronary artery remodeling. Inhibition of inflammatory responses tends to attenuate cardiac fibrosis in experimental models [10]–[13]. In DOCA/salt-induced hypertensive rats, pharmacological targeting of monocyte/macrophage accumulation ameliorated cardiac perivascular and interstitial fibrosis and reduced inflammatory marker expression, such as interleukin 6 (IL6) and monocyte chemotactic protein-1 (MCP-1) [10]–[14]. In a rat model of suprarenal aortic constriction, antibody blockade of either intercellular adhesion molecule-1 (ICAM-1) or MCP-1 reduced early macrophage recruitment, and prevented the development of myocardial fibrosis [12], [13]. However, the triggers for initiation of perivascular inflammation in the setting of LVH are poorly understood.

Accumulating evidence implicates platelets in vascular inflammation in a variety of settings. Platelets, small cytoplasmic bodies that lack nuclei, are generated by megakaryocytes in bone marrow. In settings of vascular injury or inflammation, platelets adhere to the subendothelial matrix or endothelial cells. Subsequent platelet activation maintains hemostasis through platelet to platelet interactions that form the primary platelet clot. Activated platelets express and release granule contents. Activation-dependent surface expression of adhesive molecules such as P-selectin, serve to recruit leukocytes; inflammatory mediators released by platelets may modulate leukocyte responses at sites of vascular injury [15]–[20]. Thus, in addition to their essential role in hemostasis, platelets have been proposed to serve as important mediators of vascular inflammation [15], [20]–[26].

We previously reported that pressure overload in mice created by transverse aortic constriction (TAC) is associated with a marked perivascular inflammation, reactive myocardial fibrosis, and medial thickening of intramyocardial coronary arteries [27]. We and others have observed a reduction in coronary artery flow reserve (CFR) following TAC [28]–[30], raising the possibility that acute perivascular inflammation plays a role in intramyocardial artery remodeling during LVH. In this report, we investigated early cellular events that mediate perivascular inflammation and coronary artery remodeling. We report that platelets and T lymphocytes are recruited to coronary arteries after TAC, and that both cell types may play a role in modulating perivascular inflammation and fibrosis in the setting of LV pressure overload.

## Materials and Methods

### Animals

All procedures conformed to the recommendations of “Guide for the Care and Use of Laboratory Animals" (Department of Health, Education, and Welfare publication number NIH 78-23, 1996), and were approved by the Institutional Animal Care and Use Committees at the University of Kentucky. The C57BL/6J (B6), RAG-1-deficient (Rag1^−/−^) mice, and IL-10-deficient (IL-10^−/−^) mice, were obtained from The Jackson Laboratory (Bar Harbor, ME). All the mice were maintained on the C57BL/6J background.

### Transverse aortic constriction model

**Figure 1:**
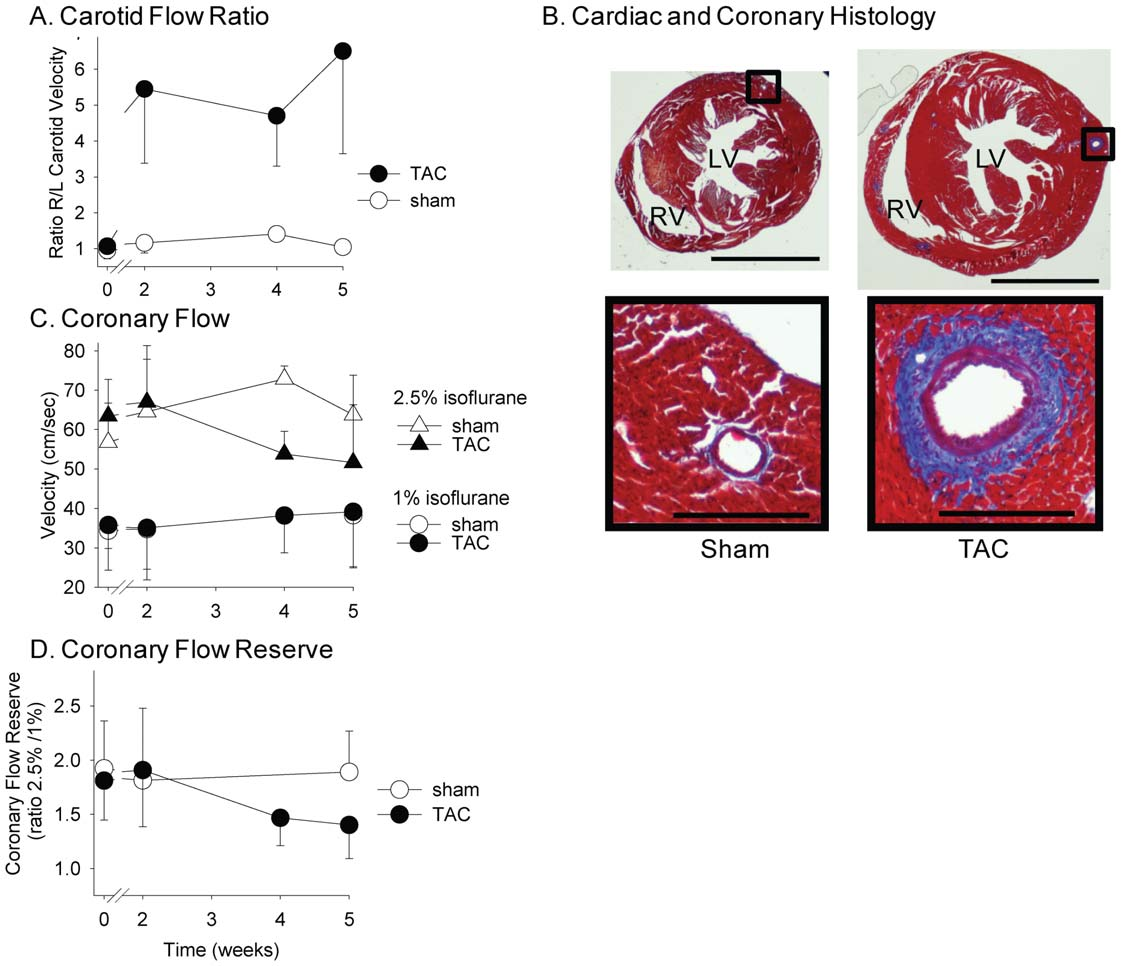
TAC-induced LV pressure overload stimulates left coronary remodeling and dysfunction in wild-type C57BL/6 male mice.

Pressure overload of the LV was elicited by TAC in male mice at the age of eight to 12 weeks. Sham surgery, in which a suture was passed around the aorta but removed without tying, was performed as a control [27]. Mice were sacrificed at early (one day, three days, and seven days) and late (five weeks) time points after surgery. Left ventricle function and coronary flow reserve were measured by echocardiography and Doppler (Figure 1).

### Doppler studies

Velocity of blood flow in the left and right carotid arteries and coronary artery was measured as described previously [29], [30] using a hand-held 20 MHz Doppler probe (Indus Instruments, Houston, TX) before and after surgery, as well as at additional time points post-surgery. The mice were anesthetized with isoflurane 1.5–2%, and taped supine to a temperature-controlled board. The board included electrocardiographic electrodes placed under each limb and a heating pad under the body. Body hair was clipped and the skin was moistened with water to improve sound transmission. The Doppler probe was placed on the left and right sides of the neck at a 45° angle to detect flow velocity in the left and right carotid arteries. For coronary flow, the probe tip was placed on the left chest at the level of the cardiac base and pointed horizontally toward the anterior basal surface of the heart to sense blood-flow velocity. The optimal Doppler flow velocity signals were obtained by adjusting the position of the probe and the Doppler range gate depth of 2 mm (for carotid artery), or 2.5–3 mm (for coronary artery), to obtain a maximal velocity signal. A computer-based Doppler signal processor (Model DSPW, Indus Instruments) was used to store the Doppler signals and for later analysis. For left coronary artery flow the baseline flow velocity was recorded at 1% isoflurane concentration. The hyperemic flow velocity was recorded during maximal vasodilatation induced by 2.5% isoflurane and after three minutes of isoflurane inhalation. Coronary artery flow reserve was calculated as the ratio of hyperemic peak flow velocity to baseline peak flow velocity.

### Echocardiography

Two-dimensional short- and long-axis views of the left ventricle were obtained by transthoracic echocardiography performed five weeks post-TAC using a 45 M-Hz probe (77B) and the Vevo 770 Imaging System (VisualSonics, Toronto, Canada), under 1.5% isoflurane inhalation as previously described [1]. M-mode tracings were recorded and used to determine LV-end diastolic diameter (LVEDD), LV-end systolic diameter (LVESD), and LV-posterior wall thickness in diastole (LVPWThD), over three cardiac cycles. Left ventricular fractional shortening (FS) was calculated using the formula % FS = (LVEDD−LVESD)/LVEDD.

### Histology

Mice were weighed and hearts, lungs, livers and kidneys were dissected at days one, three, seven and five weeks post-surgery. Hearts were harvested, washed in PBS, and sectioned at the level of the papillary muscle. The base of the heart was immersed in 4% PFA for 24 hours, subsequently transferred to 70% ethanol, and embedded in paraffin. Multiple serial 5 µm sections (six – 10) were taken every 250 µm from the distal region of the heart up the base to the aorta. Slides (six – 10) were created that contain two sections every 250 µm spanning the base of the heart. Slides from each heart were stained with hematoxylin and eosin, Masson's Trichrome and picrosirius red staining. The remaining slides were archived for later use.

Paraffin-embedded immunohistochemistry (IHC) was done with a mouse-on-mouse smooth muscle cell actin-α antibody (Sigma A-5691). In brief, heart sections were first de-paraffinized and placed in diluted antigen unmasking solution (Vector, Burlingame, CA) for antigen retrieval using a decloaking chamber (Biocare Medical, Concord, CA). Non-specific sites were blocked using mouse-on-mouse IgG blocking reagent (Vector Labs) for one hour at room temperature (RT). Slides were then incubated with primary antibody for one hour at RT, followed by 30 minutes with substrate solution (Vector Red substrate). CAT Hematoxylin (Biocare Medical) was used for counter-staining. Finally, slides were dehydrated, mounted with DPX coverslip (Gallard Schlesinger Industries, Garden City, NY) and covered by cover glasses. Positive staining was quantified using Metamorph software.

Immunohistochemistry was performed on frozen sections using CD90.2 (1∶100, BD Pharmingen™, San Diego, CA), CD68 (1∶200, Serotec, Raleigh, NC), VCAM-1(1∶50, BD Pharmingen™), PECAM-1(1∶50, BD Pharmingen™), CD8 (1∶50, Serotec), CD19 (1∶50, BD Pharmingen™), MPO (1∶50, Abcam, Cambridge, MA) and platelet antibody (1∶1000, Intercell, Gaithersburg, MD). Briefly, the heart bases were directly embedded in OCT and frozen at −20°C. Blocks were cut into 10 µm sections and fixed with chilled acetone in −20°C. To block endogenous peroxidases, slides were immersed in 1% H_2_O_2_ in methanol for two minutes at 40°C. Non-specific sites were blocked using 1.5% serum from the secondary antibody-derived animal for 15 minutes at 40°C. Slides were then incubated with primary antibodies for 15 minutes at 40°C, then for 15 minutes with biotinylated secondary antibody, and then for 10 minutes with ABC detector (Vector Labs) at 40°C. Hypersensitive response and pathogenicity substrate-chromogen (Biomed) was used as the chromogen, and hematoxylin (Accurate Chemical & Scientific Corp., Westbury, NY) was used for counter-staining. Frozen slides were fixed with chilled acetone in −20°C. Non-specific sites were blocked using 1.5% serum from the secondary antibody derived animal for 20 minutes at RT. Slides were then incubated with PECAM-1 primary antibody (1∶50, BD Pharmingen™) at RT for one hour and a rhodamine-conjugated goat-anti-rat secondary antibody (15 µg/ml, Jackson ImmunoResearch, West Grove, PA) at RT for 30 minutes. Slides were viewed and images were taken using a Leica TCS SP5 laser scanning inverted confocal microscope.

Immunostaining of targeted proteins was quantified in tissue sections from TAC mice and their respective sham controls. For each antibody, isotope-matched non reactive IgG served as the negative control. The percentage of the vessel area occupied by inflammatory cells was measured from digital images using Metamorph software by personnel blinded to treatment. Perivascular and interstitial fibrosis was measured from Masson's Trichrome and picrosirius red-stained slides. Vessel area was quantified using Metamorph software and reported as external elastic laminar.

### Quantitative PCR

RNA samples were extracted from the apex of the heart (stored in RNA later, −80°C) using TRIzol (Invitrogen, Grand Island, NY) as previously described [31]. RNA concentration and quality were tested using OD _260/280_ ratio by Biomate3 (Thermo Electron Corporation, Waltham, MA) and also by examining the product on a 1% agarose gel. The cDNA synthesis was performed using High-Capacity cDNA Archive kit (Applied Biosystems, Foster City, CA), starting with 1 µg RNA in 20 µl reaction system. Quantitative, real-time PCR was done using Taqman Universal Master Mix and Assays-on Demand primers and probes (Applied Biosystems) and the ABI 7500 system. 18S RNA was used as an endogenous control. An embryo RNA standard was used as a positive control. Results were expressed as mean fold changes of gene expression relative to sham-operated C57Bl/6 mice or control antibody-injected TAC mice using 2^−ΔΔCT^ method [31].

### Flow cytometry

Fifty µl blood was collected in 0.32% citrate and incubated with anti-Mac-1 antibody (PE conjugated, BD Pharmingen™) at a final concentration of 20 µg/ml, or rat IgG (PE conjugated, Emfret, Eibelstadt, Germany) as an isotype control in the presence of 1 µM RGDS peptide to prevent platelet aggregation. CD41 Ab (FITC conjugated, BD Pharmingen™) was added to give a final concentration of 20 µg/ml. To activate platelets, 150 µM PAR4 activating peptide (Anaspec, Fremont, CA) was added. After 15 minutes of incubation, 1 ml of Phosflow Lyse/Fix buffer solution (BD Biosciences, San Jose, CA) was added to each sample. Platelet-leukocyte aggregates were quantified using FACS caliber flow cytometer (BD Biosciences).

To measure platelet activation by flow cytometry, blood was collected into 0.32% citrate and diluted further in Tyrode's Buffer with 0.35% BSA and 1 mM MgCl_2_. The diluted blood was incubated with 60 µg/ml FITC anti-fibrinogen (DAKO Corp., Carpinteria, CA) or 20 µg/ml FITC anti-P-selectin antibody (BD Biosciences) and the indicated agonist, for 10 minutes in the dark. The reaction was stopped by the addition of Tyrode's buffer and paraformaldehyde. Samples were quantified by flow cytometer by gating on platelet populations identified by forward and side-scatter properties.

### Whole blood aggregation

Aggregation was studied in whole blood using a Multiplate®, Platelet Function Analyzer (Dynabyte, Munich, Germany). 300 µl of whole blood was diluted 1∶1 with normal saline (0.91%) and 1 mM MgCl_2_ solution and incubated for 3 minutes at 37°C, prior to the addition of 6.5 µM ADP. Aggregation units (AU) and the area under the curve (AUC in AU * minutes) was calculated based on measurements of electric impedance.

### Mouse plasma collection

Mouse blood was collected into EDTA–coated tubes, immediately centrifuged at 2000 g for 10 minutes at RT, and plasma stored at −80°C.

### Mouse cardiac tissue extraction

The base of the LV was dissected (2 mm×2 mm pieces) in 250 µl of PBS containing protease inhibitors (Pierce), homogenized using Omni tissue homogenizer, and then sonicated for 10 seconds. Following centrifugation (14000 rpm for 15 minutes at 4°C), approx 200 µl of supernatant was diluted with 200 µl PBS containing protease inhibitors.

### Luminex assay

Inflammatory cytokine/chemokine levels in mouse plasma and extracts of cardiac tissue were measured by Luminex bead analyte assay using Milliplex MAP Kit according to the manufacturer's instructions (Millipore Corp., Billerica, MA).

### Culturing and treatment of myocardial fibroblasts

Primary myocardial fibroblasts were isolated from C57BL/6 male mice by mincing hearts in1 ml of dispase solution. After fully mincing, 4 ml dispase solution was added, the sample placed at 37°C, and pipetted up and down at 15 minutes. After 30 minutes, 5 ml HBSS+ solution was added to neutralize the dispase. The cell suspension was then centrifuged at 2200 rpm for eight minutes. The cell pellet was saved and resuspended in plating medium (low glucose DMEM+10% FBS+penicillin/streptomycin). Cells from one heart were settled in one, six-well plate over night, washed the next day, and added with fresh media. The attached myocardial fibroblasts were cultured for seven to 10 days to reach a 70% to 80% confluence before treatment.

Washed platelets were isolated from ACD buffered C57BL/6 male mice blood. 1.5×10^9^ platelets were stimulated using 1 U/ml of thrombin at 37°C for 10 minutes, then neutralized with 1 U/ml hirudin. The suspension was then centrifuged at 3000 g at 4°C for 10 minutes and supernatant was collected. Myocardial fibroblasts were treated with 1 ml/well of either platelet releasate or buffer (both diluted 1∶5 in medium with 0.1% FBS). Cells were harvested at six hours or 24 hours, and RNA was isolated using RNeasy Plus mini kit (Qiagen, Valencia, CA). The cDNA synthesis and quantitative PCR were performed as described before.

### Statistical analysis

All results were expressed as mean ± standard error of the mean (SEM). Statistical significance within strains was determined using *t*-test or two-way ANOVA with multiple pair-wise comparisons, as appropriate. The Mann-Whitney test was used for nonparametic analysis. Statistical analysis was performed using Sigma-STAT software, version 3.5 (Systat Software, Inc., Chicago, IL). A *p*-value of less than 0.05 was considered significant.

## Results

### TAC is accompanied by early pericoronary inflammation

**Figure 2:**
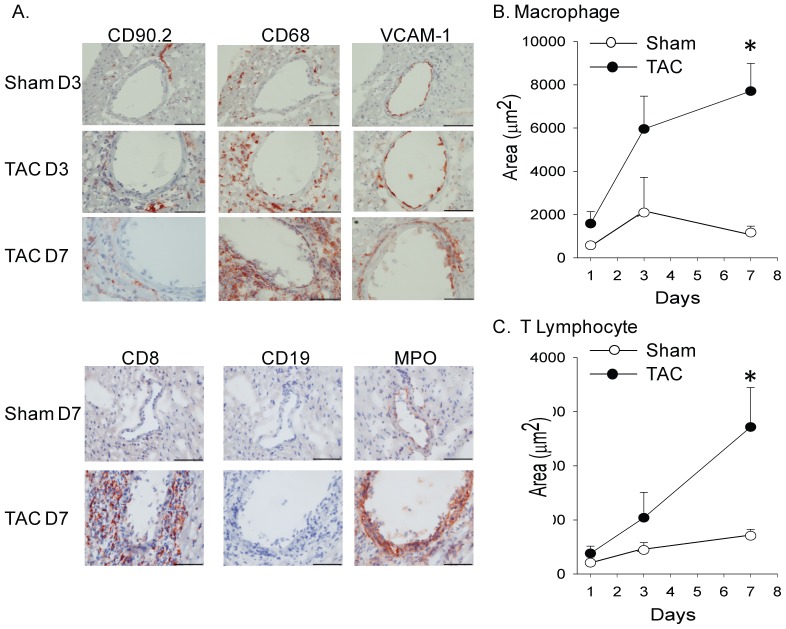
TAC elicits an early inflammatory response in wild-type (C57BL/6) male mice. A) Immunohistochemical staining of inflammatory cells and adhesion molecules in LV sections from wild-type male mice subjected to sham or TAC surgery and sacrificed at different time points (D = days). Positive staining is in red-brown (mag. 40×; Bar = 70 µm T lymphocytes are indicated by CD90.2 and CD8; CD19 is a B lymphocyte marker. VCAM is an endothelial inflammatory marker and myeloperoxidase (MPO) an enzyme stored and released by neutrophils and macrophages. B) and C) Quantification of the accumulation of macrophage (CD68) and T lymphocyte (CD90.2), as measured by area of positive staining in and around the coronary arteries, at different time points following sham (open circles) and TAC (closed circles) surgery. Values are presented as mean ± sem. The images are representative of those obtained in the following numbers. For macrophages: TAC day one, n = 5; TAC day three, n = 7; TAC day seven, n = 9; sham day one, n = 3; sham day three, n = 4; sham day seven, n = 5. For lymphocytes: TAC day one, n = 7; TAC day three, n = 6; TAC day seven, n = 6; sham day one, n = 4; sham day three; n = 5; sham day seven, n = 5. *P<0.05 versus same time point in sham.

**Figure 3:**
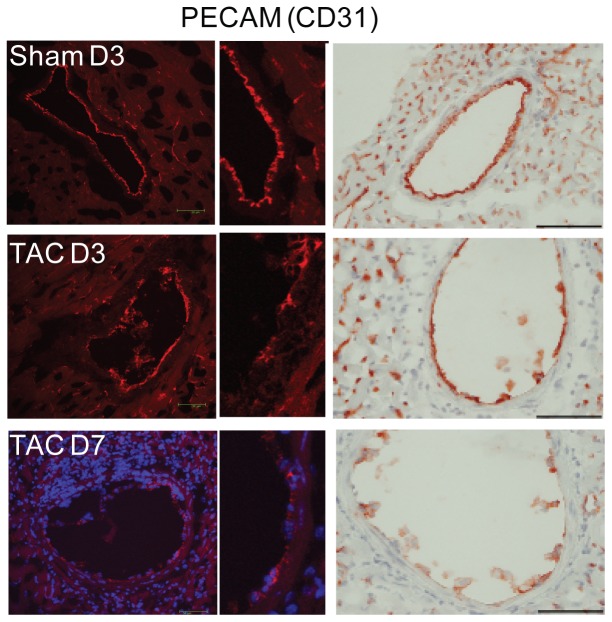
Endothelial disruption at three and seven days after TAC surgery. Confocal (left and middle panel) and IHC (right panel) images of PECAM (CD31) staining along the lumen of left coronary arteries at three days after sham surgery (sham D3) and at three (TAC D3) and seven days (TAC 7D) after TAC surgery. CD31 staining appears continuous along the luminal side in sham mice and discontinuous along similar vessels in TAC mice. Bar = 70 µm.

Transverse aortic constriction (TAC) induces an acute and persistent increase in proximal aortic and LV pressure, manifested by an elevation in the ratio of the right to left carotid peak flow velocity (Figure 1A). Through cellular and molecular events that are not completely understood, LV pressure overload in mice elicits significant perivascular and interstitial cardiac fibrosis at five weeks post-surgery [27] (Figure 1B). As reported by Hartley and colleagues [28]–[30], we observed a decline in coronary flow reserve (CFR) after TAC, indicating dysfunction of the coronary arteries (Figures 1C and 1D). In the present study, we investigated the contribution of early perivascular inflammation to coronary artery remodeling and perivascular fibrosis in the setting of pressure overload. As early as one day post-surgery, inflammatory cells accumulated along the left coronary arteries (Figure 2). Both macrophages (CD68^+^; Figures 2A and 2B) and T-lymphocytes (CD90.2^+^; Figures 2A and 2C) in the vessels increased by approximately four-fold during the first week post-TAC. The CD8 staining confirmed the presence of T cells. However, B cells stained by CD19 were not detected after TAC (Figure 2A). The expression of vascular cell adhesion molecule 1 (VCAM-1) and myeloperoxidase (MPO) were also increased after TAC (Figure 2A). The inflammatory infiltration in the left coronary arteries was accompanied by a perturbation in the endothelium, as evidenced by discontinuous platelet-endothelial cell adhesion molecule-1 (PECAM-1, CD31) staining (Figure 3).

**Figure 4:**
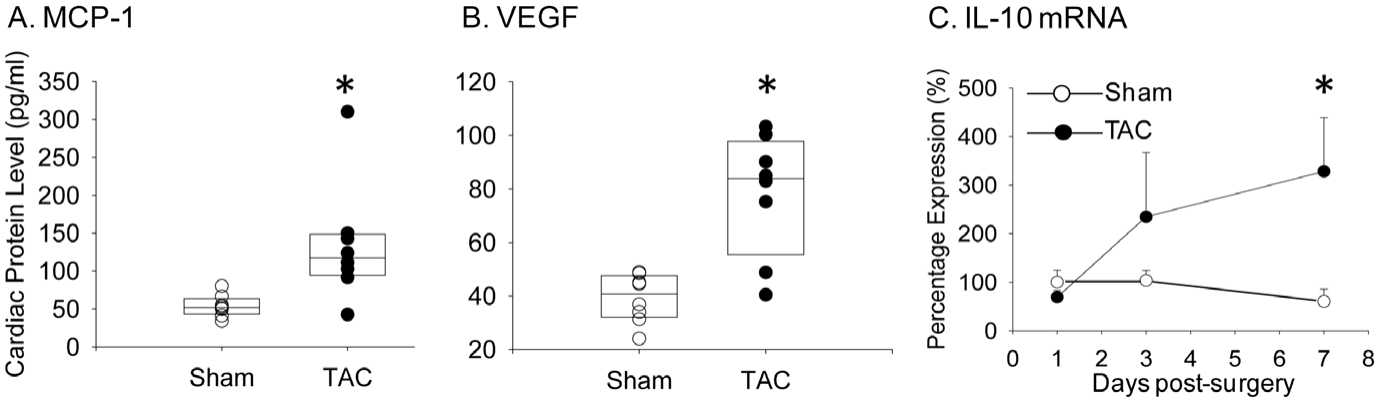
Upregulation of inflammatory markers after TAC in wild-type mice. Levels of MCP-1 (A) and VEGF (B) were measured in homogenized LV at seven days after sham (n = 6) or TAC surgery (n = 8) by Luminex assay. Protein expression (pg/ml) in the homogenate is reported for each individual mouse, and boxes indicated median and 95% CI. (C) RNA was isolated from LV apex at one to seven days after TAC (n = 3–7 per time point). IL-10 mRNA levels were measured by qPCR and values normalized to the sham value at day one, which was set at 100% expression, and presented as mean ± SD. *P<0.05 versus sham.

To provide insight into the underlying mechanisms for inflammation and remodeling early after TAC we measured RNA or protein levels of candidate cytokine mediators, such as monocyte chemotactic protein-1 (MCP-1), intercellular adhesion molecule-1 (ICAM-1), tumor necrosis factor-α (TNF-α), interleukin-6 (IL-6), which were known to contribute in other hypertrophy models [10]–[14]. We also measured levels of growth factor β-1 (TGFβ-1) [32]–[35] and vascular endothelial growth factor (VEGF), which have demonstrated roles in fibrosis and angiogenesis in the setting of cardiac hypertrophy. Additionally, because our IHC data indicated early T lymphocyte accumulation, we measured RNA or protein levels of IL-1β, IL-10, and interferon-γ(IFN-γ) in order to define the T lymphocyte subtypes involved. At seven days after TAC, MCP-1 (Figure 4A) and VEGF (Figure 4B), increased in cardiac tissue. Additionally, IL-10 mRNA expression increased more than three-fold in the LV between three and seven days after TAC (Figure 4C). In the first several days after TAC, there were no significant changes in TNF-α, ICAM-1, TGFβ-1 or IFN-γ mRNA levels in LV (data not shown). Plasma cytokines, including IL-1β, IL-10, VEGF, IFN-γ,TNF-α, and MCP-1, also did not appreciably change early after TAC, although a trend towards higher plasma IL-6 level post-TAC was noted (data not shown).

### Effects of thrombocytopenia on TAC-induced LV remodeling

**Figure 5:**
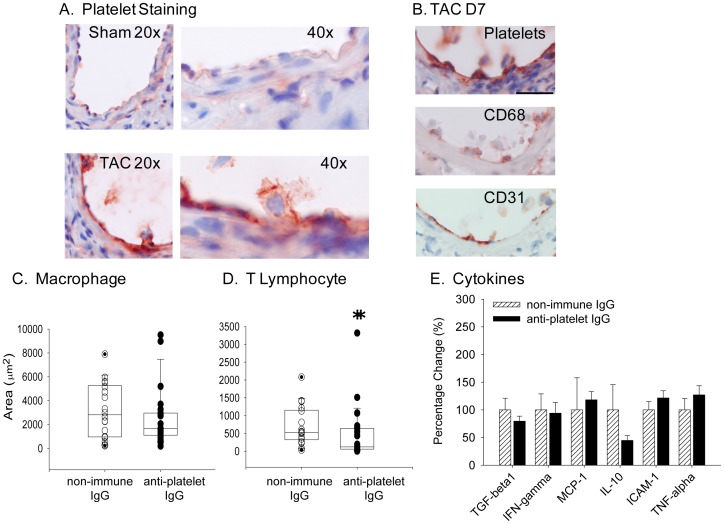
Platelet accumulation and effects on perivascular inflammation. A) Immunohistochemical staining for platelets three days after TAC (mag. 40×). B) Platelet deposition co-localized with macrophages in coronary arteries at seven days after TAC (TAC D7). Serial sections through coronary arteries were stained with antibodies to platelets (top), CD68 (middle) and CD31 (bottom). (mag. 40×; Bar = 70 µm). Area of positive perivascular staining for C) macrophages (vessel number = n, anti-GPIbα IgG n = 23, non-immune IgG n = 18) and D) T lymphocytes (vessel number = n, anti-GPIbα IgG n = 26, non-immune IgG n = 18) three days after TAC. *P = 0.05. E) Expression of inflammation markers in LV apex at three days post-TAC was measured by qPCR (mice number = n, anti-GPIbα IgG n = 13, non-immune IgG n = 10) presented as mean ± SD.

Platelets often contribute to perivascular inflammation [15], [20]–[26], and enhanced platelet activation is found in patients with cardiac hypertrophy and heart failure [16], [17]. Therefore, we investigated the contribution of platelets to the inflammatory response that accompanies TAC. At seven days after surgery, platelet counts in whole blood were higher in TAC animals (1299±226/nl) than sham mice (803±191/nl; P<0.001). Pressure overload led to platelet deposition along the damaged endothelial layer at three and seven days after TAC surgery (Figure 5A). The platelets appeared to accumulate in association with leukocytes, including macrophages (Figure 5B). Systemic platelet activation can be detected in settings of vascular inflammatory diseases, such as atherosclerosis [36]–[39]. One measure of platelet activation is the presence of circulating platelet-leukocyte aggregates in systemic circulation. In blood collected at seven days after sham surgery, 25±3% of the leukocytes had attached platelets, whereas 39±7% of the leukocytes had attached platelets in blood from TAC mice (P = 0.106). Interestingly, at three and seven days after surgery, agonist-induced platelet aggregation in whole blood and platelet P-selectin expression were lower in TAC than in sham-treated animals (data not shown), suggesting that the platelets remaining in circulation post-TAC may be less responsive to agonists.

To identify a role for platelets in TAC-induced vascular remodeling, thrombocytopenia was induced by treating mice with a glycoprotein Ibα (GPIbα) antibody that promotes platelet clearance through an Fc fragment-independent pathway [40]. Following anti-GPIbα IgG treatment, the median platelet count of 70/nL (25–75% CI: 50–101/nL) was substantially lower than the median platelet count of 1362/nL observed in mice treated with non-immune IgG (25–75% CI: 1070–1567/nL; P<0.001). White blood cell count was normal, with no lymphopenia noted after administration of anti-GPIbα IgG. The effect of antibody treatment on platelet function was confirmed by measuring tail-bleeding time. The median bleeding time was >600 seconds in mice treated with anti- GPIbα IgG (600–660 seconds; 25–75% CI) and 70 seconds (55–77 seconds; 25–75% CI) in mice treated with non-immune IgG (P<0.001). Depletion of platelets prior to TAC did not significantly alter macrophage accumulation but reduced the accumulation of T lymphocytes in the coronary arteries after TAC (from a median of 524 µm^2^ to 122 µm^2^; P = 0.031, Figures 5C and 5D). In association with lower T-cell accumulation in thrombocytopenic mice, IL-10 levels in LV were 50% lower in mice treated with anti-GPIbα IgG (Figure 5E).

**Figure 6:**
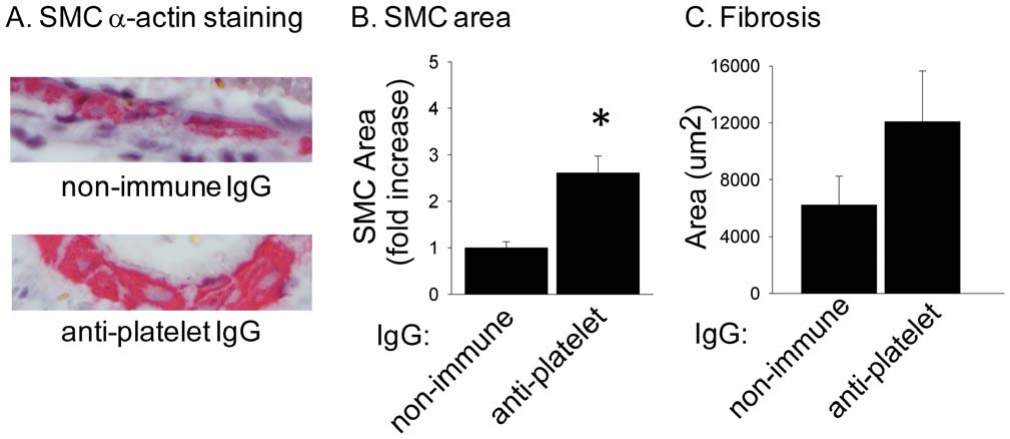
Thrombocytopenia promotes coronary vessel remodeling and perivascular fibrosis after TAC. A) Representative images (mag. 40×) and B) quantification of α-smooth muscle actin staining in coronary arteries of thrombocytopenic (anti-GPIbα IgG treated) and control (non-immune IgG injected) mice five weeks after TAC surgery (vessel number = n, anti-GPIbα IgG n = 11, non-immune IgG n = 5). *P<0.05. C) Area of pericoronary fibrosis thrombocytopenic and control mice five weeks after TAC (vessel number = n, anti-GPIbα IgG n = 10, non-immune IgG n = 5). Fibrosis was identified by Masson's Trichrome stain and reported as area mean ± sem.

To determine the long-term consequences of platelet depletion and resultant reductions in T-lymphocytes and IL-10 in the vessel wall, we examined coronary arteries at five weeks after TAC. In thrombocytopenic mice, the large coronary arteries contained significantly more smooth muscle cell (SMC) α-actin, consistent with an effect on vascular remodeling (Figures 6A and 6B). In addition, mice treated with anti-GPIbα IgG had a trend towards an increase in perivascular fibrosis (Figure 6C). These findings were not due to alterations in cardiac size or function since heart weight to body weight ratio and left ventricular internal diameter in diastole (LVIDd) posterior wall thickness (PWt), and fractional shortening (FS) (as measured by echocardiography), were similar in control and thrombocytopenic mice (data not shown).

**Figure 7:**
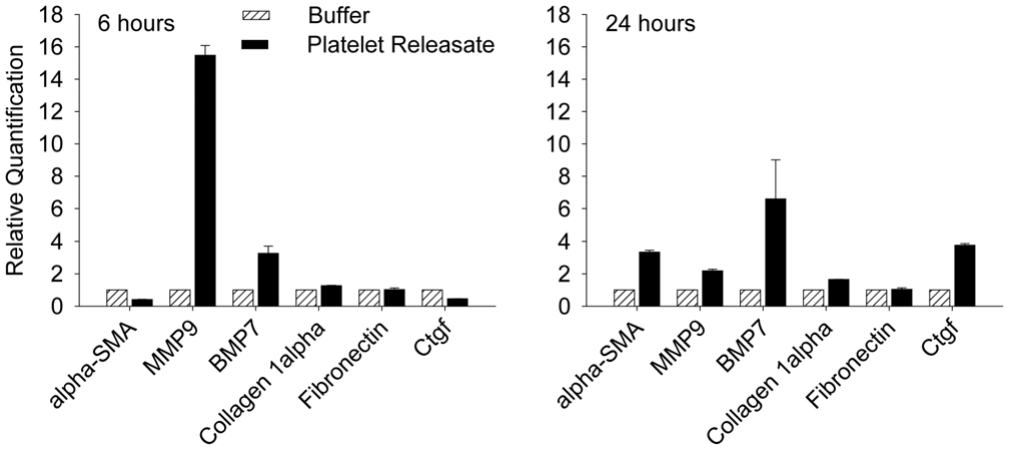
The effects of platelet releasate on myocardial fibroblasts. qPCR was performed to detect the differences of mRNA expression in cardiac fibroblasts exposed to platelet releasate (dark bars) or buffer (open bars) for six (left) or 24 (right) hours. The values were normalized to the buffer-treatment group at the same time point, which was set at fold increase and presented as mean ± SD.

In addition to recruiting inflammatory cells, platelets may release mediators that directly affect cardiac fibroblasts and thereby influence perivascular fibrosis. Therefore, the effect of platelet releasate on cardiac fibroblasts was investigated. Releasate was prepared from thrombin-activated platelets and incubated with primary cultures of murine cardiac fibroblasts. Following six hours of exposure to platelet releasate, expression of MMP9 mRNA significantly increased by 15.5±0.6-fold (Figure 7) and remained elevated at 24 hours (Figure 7). Platelet releasate also increased α-SMA expression at 24 hours. In addition, TGFβ-1 signaling appeared to be enhanced, in that the downstream targets bone morphogenic protein 7 (BMP7) and connective tissue growth factor (CTGF) were 6.6±2.4 and 3.8±0.1-fold higher following exposure to platelet releasate.

### Absence of lymphocytes exaggerates left coronary remodeling

**Figure 8:**
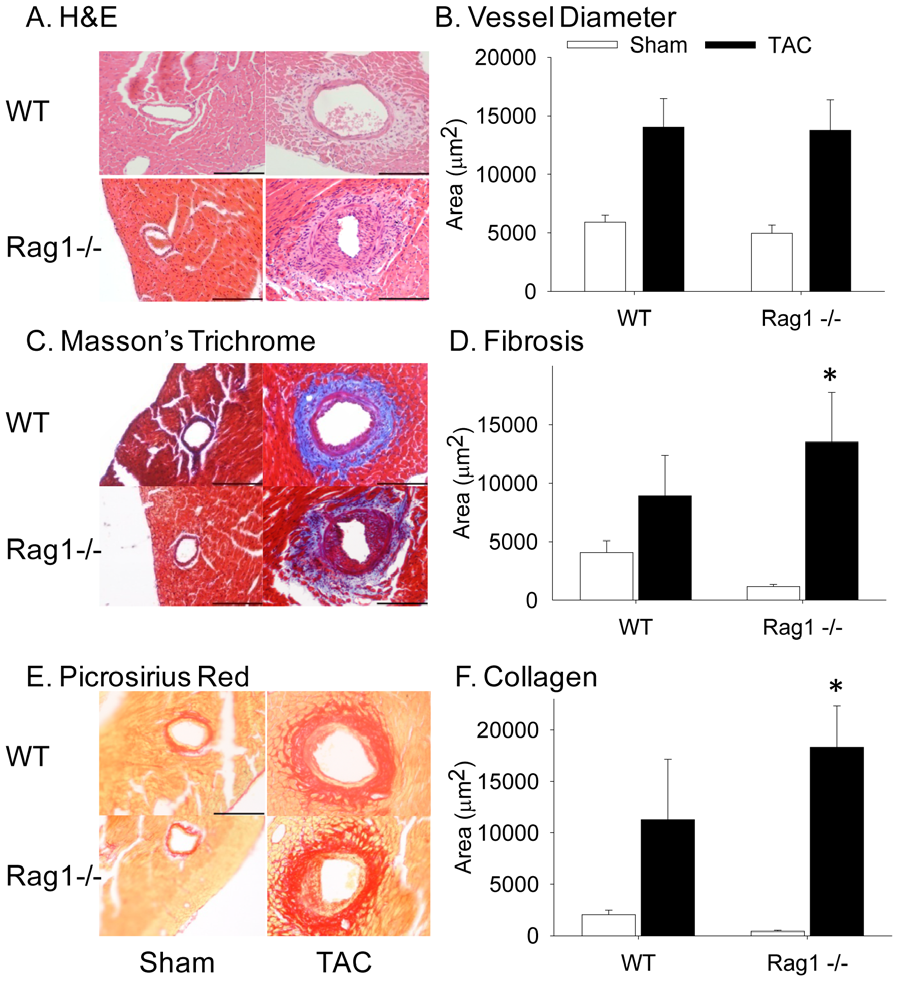
Enhanced TAC-induced coronary remodeling in the absence of lymphocytes. A) H&E staining (mag. 20×) and B) measurement of vessel area inside the external elastic laminar in wild-type (WT) and Rag1^−/−^ male mice five weeks after sham or TAC surgery (TAC: WT n = 10, Rag1^−/−^ n = 17. Sham: WT n = 9, Rag1^−/−^ n = 6). C) Masson's Trichrome staining and D) quantification of pericoronary fibrosis formation in WT and Rag1^−/−^ male mice five weeks after surgery reported as area mean ± sem (TAC: WT n = 10, Rag1^−/−^ n = 13. Sham: WT n = 9, Rag1^−/−^ n = 6). E) Picrosirius red staining and F) quantification of pericoronary collagen deposition in WT and Rag1^−/−^ male mice five weeks after surgery reported as area mean ± sem (TAC: WT n = 9, Rag1^−/−^ n = 19. Sham: WT n = 7, Rag1^−/−^ n = 7). *P<0.05. Bar = 70 µm.

Our results indicated that T (but not B), lymphocytes accrue in the vessel wall as part of the inflammatory response after TAC and that depletion of platelets reduced their perivascular accumulation. To define the impact of lymphocytes on coronary remodeling we used Rag1^−/−^ mice. *Rag1* encodes the recombination activation gene 1 that catalyzes V(D)J recombination, an essential step for the generation of immunoglobulins and T lymphocyte receptors. As a consequence, Rag1^−/−^ mice lack mature B and T lymphocytes [41]. The absence of lymphocytes in Rag1^−/−^ mice did not significantly alter the number of macrophages that accumulated in the coronary arteries seven days after TAC (data not shown). The absence of lymphocytes also did not alter the development of LVH, as HW: BW was not significantly different in wild-type and Rag1^−/−^ mice at five weeks post-TAC (Table 1). Likewise, overall coronary artery remodeling, as measured by the area of the vessel within the external elastic lamina, was similar in wild-type and Rag1^−/−^ mice after TAC (Figures 8A and 8B). However, lumen area was 1.32-fold smaller in Rag1^−/−^ coronary arteries after TAC, with a concomitant increase in collagen content. Quantification of Masson's Trichrome (Figure 8C) and picrosirius red staining (Figure 8E) indicated that Rag1^−/−^ mice had less perivascular fibrosis (Figure 8D) and collagen content (Figure 8F) at baseline but greater levels after TAC than that observed in wild-type mice. Thus, the fold increase in perivascular fibrosis (11-fold, P = 0.031, Figure 8D) and collagen content (43-fold, P = 0.008, Figure 8F) was significantly higher in Rag1^−/−^ mice post-TAC. Interestingly, despite these histological changes, the Rag1^−/−^ mice demonstrated preserved CFR after TAC. CFR was 1.83±0.15 in wild-type mice that underwent sham surgery (n = 7) and 1.72±0.23 in sham-treated Rag1^−/−^ mice (n = 3). Following TAC, CFR declined to 1.22±0.31 in wild-type mice (n = 13; P<0.05). However, CFR was not significantly different in sham and TAC Rag1^−/−^ mice (1.57±0.16; n = 7), suggesting that despite the smaller lumen size and greater collagen content, the function of the coronary arteries after TAC was preserved in the absence of lymphocytes. At baseline, in comparison to wild-type mice, Rag1^−/−^ mice had reduced LV function which did not decline further following TAC (Table 2).

**Table 1:** Organ and body weight in WT and Rag1^−/−^ mice five weeks after TAC or sham surgery.

Genotype	Treatment	n	BW (g)	HW:BW	LiW:BW	LuW:BW	KiW:BW
WT	sham	3	24.8±2.1	5.2±0.84	44.9±3.0	6.1±0.4	13.7±1.2
WT	TAC	7	25.0±1.3	6.9±0.2#	41.8±5.4	6.6±0.5	14.1±0.7
Rag1^−/−^	sham	3	29.7±1.5	5.6±0.4	52.6±1.7	5.1±0.2	12.7±1.0
Rag1^−/−^	TAC	7	27.7± 1.7	6.7±0.2*	48.7±1.1	5.5±0.2	12.6±0.5

Results are presented as mean ± sd; BW = body weight, HW = heart weight, LW = liver weight, LuW = lung weight, KiW = kidney weight. Two-way ANOVA statistics show significant differences in the ration of heart weight to body weight between TAC and sham groups in B6 and Rag1^−/−^ mice.

# P<0.001,

* P<0.05 vs sham controls.

**Table 2:** Echocardiographic analysis of heart size and function in WT and Rag1^−/−^ mice five weeks after TAC or sham surgery.

genotype	treatment	n	FS (%)
WT	sham	3	39±1.8
WT	TAC	7	25.3±1.2*
Rag1^−/−^	sham	3	32.2±1.8#
Rag1^−/−^	TAC	7	28.4±1.8

FS = fractional shortening;

* P<0.05;

# P<0.001 vs WT sham control.

### IL-10 may play a protective role in LV remodeling

**Figure 9:**
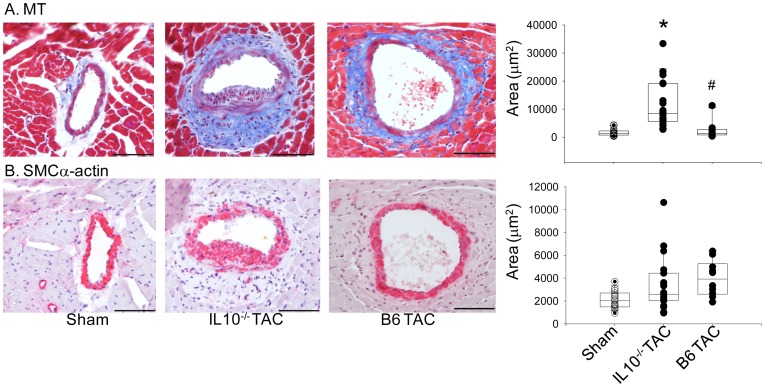
TAC-induced coronary remodeling in the absence of IL-10. A) Representative sections stained with Masson's Trichrome (MT) and visualized at 40× mag. five weeks after sham or TAC surgery (left) and measurements of perivascular fibrosis area (sham n = 5; TAC n = 5). B) SMC α-actin staining five weeks after sham or TAC surgery (left) and measurements of vessel area occupied by SMC α-actin (right). *P<0.05. Bar = 70 µm.

Platelets can influence the function of T-cells by eliciting the release of IL-10 from dendritic cells [42]. The finding that thrombocytopenia lowered both T-cell infiltration and IL-10 levels after TAC suggested that platelets may be influencing T-cell-mediated events in an IL-10-dependent manner in this model. Therefore, we sought to determine if IL-10 deficiency recapitulated aspects of the phenotype observed in thrombocytopenic or lymphopenic mice. Cardiac hypertrophy, as assessed by HW: BW ratio, was not significantly different in IL-10^−/−^ and wild-type mice after TAC (7.7±2.0 and 7.1±1.3, respectively, P = 0.596). However, IL-10^−/−^ LV displayed substantial perivascular fibrosis as detected by Masson's Trichrome staining (Figure 9A) with levels (11,600±2000 µm^2^, mean ± sem; n = 5) similar to those observed in thrombocytopenic and Rag1^−/−^ mice post-TAC, and was significantly higher than B6 mice. The IL-10^−/−^ TAC mice also displayed evidence of exaggerated remodeling as measured by SMC α-actin-staining, with a similar level of B6 mice (Figure 9B).

## Discussion

We previously reported that pressure overload in mice elicited significant perivascular and interstitial cardiac fibrosis at five weeks post-surgery [27]. As reported by Hartley and colleagues, we observed impaired coronary artery function, as measured by CFR, after TAC [28]–[30]. In the present study, we expanded our findings by examining the contribution of early perivascular inflammation to coronary artery remodeling in the setting of pressure overload. A striking and rapid accumulation of platelets, macrophages, and T lymphocytes occurred around the left coronary vessels post-TAC. Quantification of coronary vessel size revealed that TAC mice had a significant increase in vessel size as compared to sham-operated animals. In parallel, perivascular fibrosis formation significantly increased. Although the precise mechanism(s) by which changes in flow may affect the coronary remodeling after TAC are unknown, numerous studies have shown that flow-induced changes in shear stress can contribute to perivascular inflammatory responses in coronary atherosclerosis and in-stent restenosis [43], [44]. In particular, PECAM-1 (CD31) serves as part of the mechanical sensor complex regulating flow-induced vascular inflammation and remodeling [45]–[49]. In our model, endothelial disturbance, as evidenced by discontinuous PECAM staining, occurred within three days after TAC. Therefore, we hypothesize that pressure overload in the left ventricle alters coronary flow, shear stress, and endothelial function, which subsequently triggers platelet deposition and perivascular accumulation of inflammatory cells. The precise molecular mechanism(s) responsible for the changes are presently not understood. Future studies will investigate whether PECAM-1 expression or phosphorylation is altered by flow changes resulting from TAC.

The appearance of platelet positive staining on the luminal surface of left coronary vessels and co-localization with leukocytes, suggested a role for platelets in coronary artery remodeling in response to pressure overload. Previous literature has reported that platelets dynamically influence the immune system through CD154 (CD40L) mediated interactions with CD40 on T and B lymphocytes [50]–[52]. Moreover, platelets enhance IL-10 expression by immature dendritic cells, which in turn promote the proliferation of naive T cells [42]. We report that pericoronary T lymphocytes accumulation was significantly reduced in thrombocytopenic mice, suggesting a potential role for platelets in T lymphocytes recruitment, either through direct contact or indirectly through mediators. Our immunodepletion protocol resulted in thrombocytopenia for up to one week, supporting a role for platelets in this time frame. Because platelet deposition and lymphocyte accumulation appeared as early as 24 hours after TAC (data not shown and Figure 2A), we are unable to establish whether platelet deposition is the initiating event or whether it occurs in response to inflammatory changes and serves to perpetuate the inflammatory response.

Exposure to high shear stress enables platelets to elicit IL-10 production from dendritic cells [42]. The change in flow following TAC may trigger a similar response from platelets and contribute to IL-10 expression and T-cell accumulation, which would account for lower levels of IL-10 and T cells in thrombocytopenic animals. The subsequent increase in SMC α-actin in thrombocytopenic animals could reflect an impact of these events on either the proliferative or inflammatory profile of SMC. Immune-mediated thrombocytopenia is followed by reactive thrombocytosis. Therefore, in the anti-GPIbα IgG-treated mice, it is possible that a subsequent increase in platelet count is influencing subsequent events, although given the propensity for platelet surfaces to passivate over time, this seems less likely than an effect of the initial thrombocytopenia.

Previous reports have implicated T lymphocytes in hypertensive cardiovascular diseases [53]. Our observations in Rag1^−/−^ mice additionally suggest a role for lymphocytes in influencing remodeling by limiting neointima formation and perivascular fibrosis following TAC. Interestingly, despite the increase in fibrosis, CFR was improved in the absence of lymphocytes. These effects are likely mediated largely by T cells, as we were unable to detect changes in B-cell accumulation in cardiac tissue post-TAC. In addition, at baseline, the collagen content in Rag1^−/−^ mice was lower, supporting a role for lymphocytes in extracellular matrix dynamics under basal conditions. Others have reported lower basal vascular superoxide levels in Rag1^−/−^ mice [53]. Vascular superoxide correlates with and may influence fibrotic remodeling, and may underlie the differences in the perivascular collagen content we observed in the sham-operated Rag1^−/−^ mice.

The subsets of T cells involved in TAC-induced remodeling responses are presently unknown. Subsets of T lymphocytes appear to differentially influence pathological remodeling. For example, whereas CD1d-positive natural killer cells seem to promote the development of neointima after vascular injury in a carotid collar model [54], CD4^+^CD25^+^ regulatory T cells adoptive transfer not only reduces angiotensin-induced early infiltration of inflammatory cells in cardiac tissue, but also blunts the subsequent development of cardiac hypertrophy, fibrosis formation, and arrhythmogenic potential [55]. Moreover, a balance between T helper 1 (Th1) and Th2 cells has been proposed to contribute to coronary and myocardial inflammation, and may be essential for cardiac extracellular matrix remodeling [56]–[58]. In an immune modulation mouse model, induction of Th1 responses led to increased collagen deposition, more LV stiffness, and down-regulation of MMP-9 and MMP-13 gene expression [57].

In the TAC model, we observed a significant increase in expression of IL-10 in cardiac tissue between days one and seven post-TAC. IL-10 is a Th2 cytokine produced by many cell types, including macrophages and T helper 2 cells. IL-10 signals through the JAK/STAT3 pathway to modulate the expression of certain pro-inflammatory molecules and usually plays an important role in anti-inflammatory responses. For example, IL-10 is able to attenuate the NF-κB pathway through inhibition of IKK or NF-κB nuclear translocation [59]. Also, IL-10 can suppress vascular smooth muscle cell activation and proliferation and thereby attenuate neointima formation [15], [60], [61]. Recently, Krishnamurthy et al. reported that IL-10 treatment reduced macrophage accumulation and inflammatory cytokine expression, leading to attenuated LV remodeling after myocardial infarction [62]. In keeping with these observations, bone marrow, mononuclear cell-derived IL-10, attenuates T-cell infiltration and cardiac remodeling after myocardial infarction, which prevents cardiac dysfunction [63]. In our model, increased expression of IL-10 parallels T lymphocyte infiltration early after TAC and may serve to attenuate the inflammatory responses and suppress SMC proliferation. Indeed, lower IL-10 levels correlate with increased medial hypertrophy in thrombocytopenic mice after TAC. Likewise, IL-10^−/−^ mice display normal cardiac hypertrophy, but exaggerated vascular fibrotic remodeling post-TAC.

The results presented above indicate that platelets may influence coronary remodeling and perivascular fibrosis after TAC through recruitment of T lymphocytes and stimulating IL-10-dependent signaling. In addition, platelets may have direct effects on coronary fibroblast function by releasing mediators that phenotypically modify fibroblasts. Indeed, we found that platelet releasate directly stimulates cardiac fibroblasts to increase the MMP9 expression and upregulate TGFβ-1 signaling. Platelets contain large storage of TGFβ-1 in their α-granules that could potentially activate fibroblasts and increase MMP production. Whether platelet-triggered MMP9 production attenuates or otherwise affects fibrosis remains to be established.

**Figure 10:**
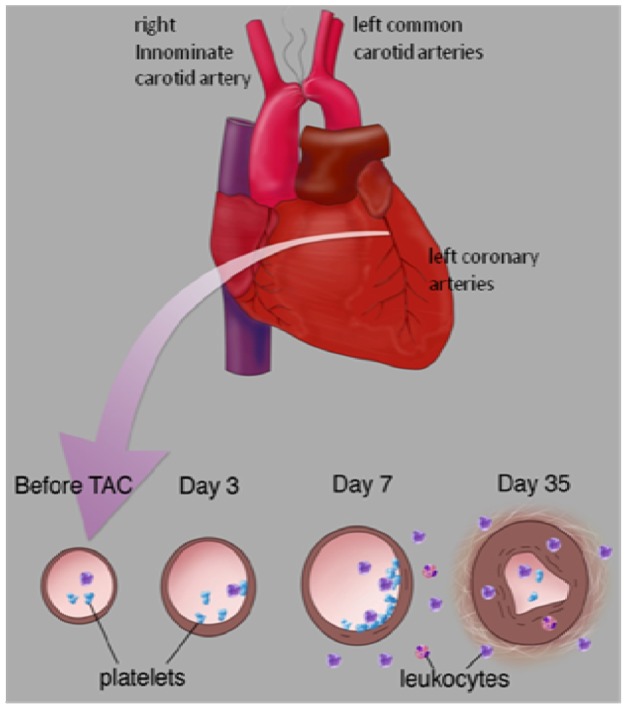
Model of TAC-induced early inflammatory response in which platelets and inflammatory cells are recruited and contribute to the subsequent development of intimal hyperplasia and fibrosis.

In summary, perivascular inflammation proceeds and accompanies coronary remodeling in the setting of pressure overload. Platelets, macrophages and lymphocytes accumulate along and around the coronary arteries early after TAC, accompanied by up-regulation of IL-10 expression in the myocardial tissue. Following TAC, the vessels undergo positive remodeling that is accompanied by extensive perivascular fibrosis (Figure 10). Surprisingly, both platelets and lymphocytes appear to play a protective role in minimizing remodeling and fibrosis. Our data are consistent with a model in which platelets influence lymphocyte recruitment – either by direct interaction or indirectly through stimulation of IL-10 (and/or other mediators) – by recruited or resident cells. The IL-10 may act to suppress ongoing inflammation and limit perivascular fibrosis. Similar mechanisms may occur in settings of hypertension and our model may have particular relevance to hypertensive urgency, in which acute and dramatic elevations in blood pressure may be associated with a disruption in endothelial function and a pro-inflammatory state in coronary arteries. The optimal strategy for minimizing coronary artery damage in the setting of acute elevations in pressure remains to be determined.
